# Direct exposure to vinclozolin induces sex-dependent mitochondrial dysfunction in mouse liver

**DOI:** 10.1016/j.crtox.2026.100302

**Published:** 2026-05-27

**Authors:** Hwayeon Lim, Jisun Choi, Joo Young Huh, Hoonsung Cho, Sooim Shin

**Affiliations:** a Department of Biomedical Engineering, The Ohio State University, Columbus, OH 43210, USA; b Department of Biotechnology and Bioengineering, Chonnam National University, Gwangju 61186, South Korea; c Department of Biomaterials Convergence, Chonnam National University, Gwangju 61186, South Korea; d College of Pharmacy, Chung-Ang University, Seoul 06974, South Korea; e Department of Global Innovative Drugs, The Graduate School of Chung-Ang University, Seoul 06974, South Korea; f Department of Materials Science & Engineering, Chonnam National University, Gwangju 61186, South Korea; g Institute of Synthetic Biology for Carbon Neutralization, Chonnam National University, Gwangju 61186, South Korea

**Keywords:** Vinclozolin, Fungicide, Endocrine disruptor, Mitochondria, Mitochondrial dysfunction, Oxidative stress

## Abstract

Vinclozolin is a dicarboximide anti-androgenic fungicide that has endocrine-disrupting impact on mammals. Endocrine disruptors are reported to impair mitochondrial function and reduce adenosine triphosphate (ATP) production. Moreover, increasing evidence has linked exposure to endocrine disruptors with metabolic diseases. However, the toxicity of direct exposure to vinclozolin on mitochondrial function remains insufficiently explored. In this study, mitochondria were isolated from mouse liver—one of the primary organs involved in the uptake and processing of toxicants—and directly exposed to vinclozolin. Several markers, including citrate synthase, mitochondrial complex IV (CIV) activity, ATP production, reactive oxygen species/reactive nitrogen species (ROS/RNS) levels, cytochrome *c* release, glutathione (GSH) levels, and superoxide dismutase (SOD) activity were measured. The results indicated that vinclozolin decreased citrate synthase activity in both sexes and significantly reduced CIV activity only in males. ATP levels showed a decreasing tendency, while ROS/RNS levels showed an increasing tendency particularly in males, without statistical significance. Notably, SOD activity exhibited a sex-dependent increase specifically in females, whereas vinclozolin exposure did not significantly alter GSH levels in either sex. Despite this, basal GSH levels remained significantly higher in females than in males. In both sexes, an increase in cytochrome *c* release was observed. Collectively, direct mitochondrial exposure to vinclozolin induced dysfunction by impairing energy production, a process mediated by sex-specific antioxidant responses.

## Highlights

• Vinclozolin impaired mitochondrial function in isolated mitochondria, reducing citrate synthase activity and increasing cytochrome *c* release.
• Mitochondrial dysfunction was more severe in males than in females.
• Females showed stronger antioxidant responses, including increased SOD activity and higher basal GSH levels.

## Graphical abstract

**Figure:**
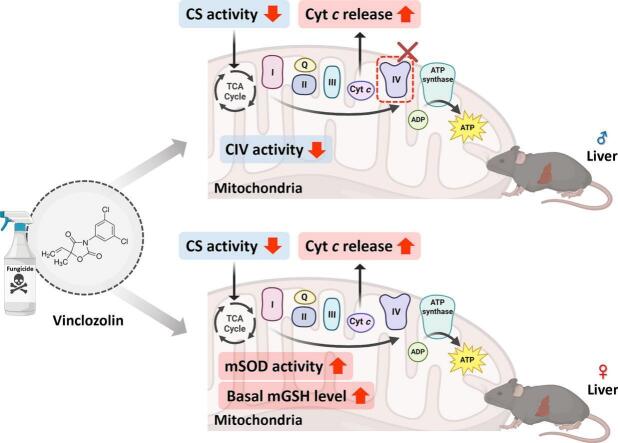

## 1 Introduction

Vinclozolin is a dicarboximide fungicide used to prevent fruits and vegetables from rotting and molding. It was widely applied in Europe and the United States until its use was banned in 2006 (Auxietre et al., 2014). However, vinclozolin and its metabolites are moderately persistent in agricultural soils, with reported half-lives ranging from 179 to 1000 days (WHO, 2013). In addition to its environmental persistence, vinclozolin is known to exhibit anti-androgenic activity, interfering with androgen receptor signaling and disrupting endocrine functions (Kelce et al., 1994). Studies have shown that fungicides can circulate through water and air, and vinclozolin may also be transferred via volatilization (Baumeister et al., 2002). Thus, vinclozolin remains of interest for experimental toxicological studies.

As a result of vinclozolin's hormonal activity, it is classified as an endocrine-disrupting compound that interferes with the hormonal regulation (Molina-Molina et al., 2006). Endocrine disruptors (EDs) have emerged as a major public health concern due to their potential to cause adverse physiological effects in humans (Yilmaz et al., 2020). These compounds disrupt hormonal signaling pathways and are associated with reproductive, immune, and neurological dysfunction (Stathori et al., 2024). Experimental studies have shown that vinclozolin exposure induces cleft phallus accompanied by hypospadias, nipple retention, epididymal granulomas, and reduced sex accessory gland weights in male rat offspring, all of which are linked to impaired reproductive function and sex differentiation (Gray et al., 1994).

Although such endocrine-mediated effects are commonly attributed to endocrine disruption, it remains possible that vinclozolin may exert additional toxic effects beyond endocrine-mediated pathways. Indeed, previous studies have reported that EDs can impair mitochondrial enzymatic function and cellular energy metabolism, leading to reduced adenosine triphosphate (ATP) production and oxidative stress (Nadal et al., 2017). Therefore, evaluating the direct toxicity of vinclozolin on mitochondrial function, independent of endocrine signaling and cellular regulation, is necessary to better understand its intrinsic toxicological properties.

Mitochondria are organelles that produce ATP as an energy source in cells by transferring electrons through the electron transport chain. They also play essential roles in reactive oxygen species generation, cell signaling, and apoptosis (Petrakis et al., 2017). Given these roles, mitochondrial dysfunction impairs energy homeostasis and contributes to the development of various severe metabolic diseases (Lowell and Shulman, 2005). Therefore, mitochondrial function has been widely used as a biomarker to evaluate its correlation with metabolic diseases. As a result, most previous studies have investigated the effects of vinclozolin on whole-cell or in vivo systems (Di Paola et al., 2022; Marcoccia et al., 2022). Several studies have also identified mitochondria as a key target of EDs, establishing a mechanistic link among EDs exposure, mitochondrial dysfunction, and metabolic disorders (Marroqui et al., 2018; He et al., 2024; Lința et al., 2024). However, there is a lack of studies investigating mitochondrial energy metabolism following the direct exposure of isolated mitochondria to vinclozolin.

Although vinclozolin is recognized as an ED, this study focuses on its direct effects on isolated mitochondria, independent of systemic hormonal regulation. Notably, mitochondria themselves exhibit sex-dependent differences in structure and function, including antioxidant capacity, membrane integrity, and susceptibility to toxicants. Previous studies have reported sex-dependent differences in mitochondrial antioxidant systems, including superoxide dismutase (SOD) and glutathione (GSH)-related pathways. In addition, oxidative stress, primarily induced by inhibition of mitochondrial complexes, is regulated by these antioxidant systems, with evidence suggesting that females possess a stronger antioxidant capacity than males (Borrás et al., 2003; Torrens-Mas et al., 2020; Iboleon-Jimenez et al., 2025). Moreover, sex-dependent differences in mitochondrial membrane properties and responses to toxicants have also been reported (Hertel Ribeiro et al., 2016; Scott et al., 2022). Therefore, considering these intrinsic differences, it is essential to evaluate mitochondrial responses separately in males and females.

The effects of EDs on the liver have been investigated, with evidence suggesting their involvement in the development of insulin resistance and type 2 diabetes (Marroqui et al., 2018). ED exposure generally induces hepatocyte injury, as indicated by alterations in sensitive markers such as aspartate aminotransferase and alanine aminotransferase (Moon et al., 2012). Additionally, EDs disrupt lipid homeostasis, leading to abnormal hepatic lipid accumulation (Huc et al., 2012), which can contribute to steatosis and the progression to hepatic carcinoma (Foulds et al., 2017). The main mechanisms underlying hepatic damage have been attributed to oxidative stress, mitochondrial dysfunction, and endoplasmic reticulum stress (Abdulhameed et al., 2022). Among these mechanisms, mitochondrial dysfunction has been associated with impaired energy metabolism, increased oxidative stress, and disrupted insulin signaling, all of which contribute to insulin resistance and type 2 diabetes. However, despite vinclozolin being one of the most extensively studied EDs, its direct effects on hepatic mitochondrial function remain poorly studied. Thus, the direct mitochondrial toxicity of vinclozolin, independent of cellular regulation, was assessed to elucidate its acute impact on hepatic mitochondria in mice. To examine mitochondrial dysfunction induced by direct exposure to vinclozolin, citrate synthase activity, a key regulatory enzyme of the tricarboxylic acid (TCA) cycle, was assessed to evaluate mitochondrial metabolic capacity, and mitochondrial complex IV activity was analyzed to evaluate oxidative phosphorylation (OXPHOS) function. In addition, reactive oxygen species/reactive nitrogen species (ROS/RNS) levels, mitochondrial antioxidant systems such as GSH levels and SOD activity and cytochrome *c* release were measured to confirm whether vinclozolin induces oxidative stress. Identifying the effects of vinclozolin on hepatic mitochondrial metabolism in both male and female mice provides insights into its relationship with metabolic disease and supports the use of mitochondria as a biomarker for evaluating the hepatotoxicity of EDs.

## 2 Materials and methods

### 2.1 Mitochondria isolation

C57BL/6 male mice (Orient Bio Inc.; Seongnam, South Korea) were housed at 22 ± 2 °C, 50–60% humidity, and under a 12 h light/12 h dark cycle in a pathogen-free room. Livers were dissected from ten-week-old male and female mice and homogenized using a tacoPrep Bead Beater (GeneReach Biotechnology Corporation, Taiwan). Mitochondria were isolated from the liver homogenates using a Mitochondria Isolation Kit for Tissue (Thermo Fisher Scientific, Waltham, USA) with a modified protocol. Briefly, 800 μL of Reagent A was mixed with the homogenates by vortexing and incubated on ice for 2 min. Next, 10 μL of Reagent B was added and vortexed for 5 s, repeated five times. Subsequently, 800 μL of Reagent C was added, and the sample was gently inverted to mix. The mixture was centrifuged at 700 ×*g* for 10 min, and the supernatant was transferred to a new 1.6 mL centrifuge tube. The sample was then centrifuged at 3000 ×*g* for 15 min, and the supernatant was collected as the cytosolic fraction. The resulting pellet was washed with 500 μL of wash buffer and centrifuged at 12,000 ×*g* for 5 min. After removing the supernatant, the pellet was resuspended in phosphate-buffered saline (PBS, pH 7.2) and stored at −80 °C for further experiments. Animal experiments were approved by the Institutional Animal Care and Use Committee (IACUC) of Chonnam National University.

### 2.2 Vinclozolin exposure to isolated mitochondria

Vinclozolin (3-(3,5-dichlorophenyl)-5-methyl-5-vinyloxazolidine-2,4-dione; CAS No. 50471–44-8; Cat. No. 45705, Sigma-Aldrich) was dissolved in dimethyl sulfoxide (DMSO) to prepare a 100 mM stock solution. The vinclozolin stock solution was diluted directly into the mitochondrial suspension in PBS to achieve final concentrations of 10, 20, 30, 40, 50, 70, and 100 μM. Isolated mitochondria were directly exposed to vinclozolin for 1 h at 37 °C.

### 2.3 Measurement of citrate synthase activity

Mitochondrial citrate synthase activity was measured spectrophotometrically following the method described by (Spinazzi et al., 2012). Briefly, 1 μg of isolated mitochondria was mixed with 100 μM 5,5′-dithiobis-(2-nitrobenzoic acid) (DTNB) (ε = 13.6 mmol^−1^ cm^−1^), 300 μM acetyl CoA, and 0.1% (*v*/v) Triton X-100 in 100 mM Tris buffer (pH 8.0). The reaction was initiated by adding 500 μM oxaloacetic acid, and the change in absorbance induced by DTNB reduction was monitored at 412 nm using an S-3100 spectrophotometer (Scinco, Seoul, Korea). The enzymatic activity was calculated using the following Equation:

(1) $$\text{Enzyme activity}\;\left(\mathrm{\mu M}\;\sec^{-1}\right)=\left(\text{ΔAbs}\;\sec^{-1}\right)/\left(\text{extinction coefficient},ɛ\right)$$

where △Abs sec^−1^ represents the slope generated by the sample.

### 2.4 Measurement of mitochondrial complex IV activity

Mitochondrial complex IV activity was measured using the method described by Spinazzi et al. (Spinazzi et al., 2012). Cytochrome *c* was reduced by sodium dithionite, and 1 μg of isolated mitochondria was mixed with 60 μM reduced cytochrome *c* in 100 mM potassium phosphate buffer. The change in absorbance induced by the cytochrome *c* oxidation was monitored at 550 nm for 600 s at 10-s intervals using SpectraMax iD3 microplate reader (Molecular Devices, USA). The complex IV activity was calculated using Eq. (1).

### 2.5 Measurement of ATP production

ATP production was measured using the ATP Determination Kit (Thermo Fisher Scientific) following a modified protocol. To induce ATP production, 2 μg of isolated mitochondria was mixed with 1 mM pyruvate and malate, 2 mM adenosine diphosphate (ADP), firefly recombinant luciferase, luciferin, and dithiothreitol (DTT). The luminescence induced by the luciferin–ATP reaction was measured using SpectraMax iD3 microplate reader (Molecular Devices, USA).

### 2.6 Measurement of mitochondrial reactive oxygen species/reactive nitrogen species (mROS/RNS) levels

Mitochondrial ROS/RNS levels were measured using the OxiSelect In Vitro ROS/RNS Assay Kit (Green Fluorescence) (CELL BIOLABS, Inc., USA) following a modified protocol. Briefly, 3 μg of isolated mitochondria was mixed with 50 μL of catalyst and incubated at room temperature for 5 min. Subsequently, 100 μL of dichlorodihydrofluorescin (DCFH) solution was added, and the mixture was incubated for 15 min at room temperature. The fluorescence induced by DCFH oxidation in response to ROS/RNS was measured using SpectraMax iD3 microplate reader (Molecular Devices, USA).

### 2.7 Measurement of mitochondrial glutathione (mGSH) levels

mGSH levels were determined using the Glutathione Assay kit (Sigma-Aldrich, USA) following a modified protocol. In this assay, mGSH reduced DTNB to TNB, which exhibits absorbance at 412 nm. Briefly, 2 μg of isolated mitochondria was mixed with 150 μL of working solution and incubated at room temperature for 5 min. The reaction was initiated by adding 50 μL of NADPH, and the change in absorbance induced by TNB formation was measured using SpectraMax iD3 microplate reader (Molecular Devices, USA). The GSH levels were calculated using Eq. (2).

(2) $$\text{nmoles}\;\mathrm{GSH}\;\mathrm{per}\;\mathrm{mL}\;\text{of sample}=\frac{△\mathrm{Abs}412\;\min\left(\text{sample}\right)-1\times \text{dilution factor of sample}}{△\mathrm{Abs}412\;\min\left(1\;\text{nmole}\;\mathrm{GSH}\right)-1\times \text{volume of sample in the reaction in}\;\mathrm{mL}}$$

where △Abs_412_/min(sample) represents the slope generated by the sample, and △Abs_412_/min(1 nmole GSH) denotes the slope obtained from the standard curve for 1 nmole of GSH.

### 2.8 Measurement of mitochondrial superoxide dismutase (mSOD) activity

mSOD activity was determined using the Superoxide Dismutase Activity Assay kit (Abcam, UK) following a modified protocol. Briefly, 2 μg of isolated mitochondria was mixed with 150 μL of Water Soluble Tetrazolium (WST) solution, and the change in absorbance was measured using SpectraMax iD3 microplate reader (Molecular Devices, USA). In this assay, superoxide radicals were reduced by mSOD or WST-1 in a competitive reaction, resulting in the formation of WST-1 formazan, which exhibits absorbance at 450 nm. The mSOD activity was expressed as the inhibition rate of WST-1 formazan formation and calculated according to Eq. (3). Each absorbance was subtracted from the initial absorbance.

(3) $$\text{mSOD activity}\;\left(\text{inhibition rate}\%\right)=\frac{\left(\text{Ablank}1–\text{Ablank}3\right)–\left(\text{Asample}–\text{Ablank}2\right)}{\left(\text{Ablank}1–\text{Ablank}3\right)}\times 100\left(\%\right)$$

where A_sample_ is the change in absorbance of the sample, A_blank1_ is the absorbance without SOD, A_blank2_ is the sample blank, and A_blank3_ is the reagent blank.

### 2.9 Measurement of cytochrome *c* (cyt c) release

Cytochrome *c* release assay was conducted by Quantikine ELISA Rat/Mouse Cytochrome *c* Immunoassay Kit (R&D system, USA&Canada). 1 μg mitochondria were incubated with 0.5% (*v*/v) Triton-X 100 in PBS (pH 7.2) for 10 min at 4 °C to solubilize the mitochondrial membrane. The sample was centrifuged at 16,000 ×*g* for 10 min. The supernatant was transferred to the 96 well plates provided in the kit. 75 μL Rat/Mouse Cytochrome *c* Conjugate was added and incubated for 2 h at room temperature. Each well was washed by 400 μL Wash Buffer, and 100 μL Substrate Solution was added. After incubation for 30 min at room temperature, 100 μL Stop Solution was added. The absorbance caused by the oxidation of tetramethylbenzidine was measured at 450 nm by SpectraMax iD3 microplate reader (Molecular Devices, USA).

### 2.10 Statistical analysis

Statistical analysis was performed using GraphPad Prism 8.0.1 software (GraphPad, Inc., La Jolla, CA, USA). Data are presented as mean ± standard deviation (SD). Statistical significance was evaluated using two-way analysis of variance (ANOVA) followed by Dunnett's multiple comparison test or an unpaired *t*-test, as appropriate.

## 3 Results

### 3.1 Vinclozolin decreased citrate synthase activity and reduced mitochondrial complex IV activity, especially in males

**Fig. 1:**
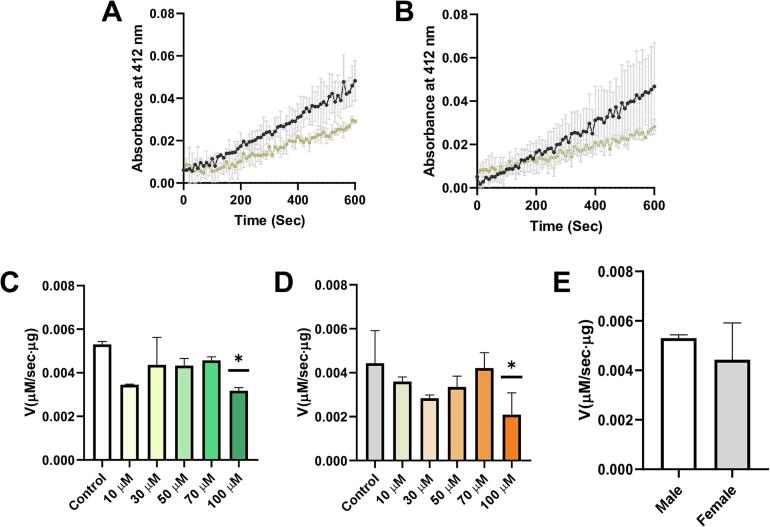
Citrate synthase activity after vinclozolin exposure on mouse liver. Citrate synthase activity after vinclozolin exposure. The change in absorbance of TNB in response to 100 μM vinclozolin exposure was monitored at 412 nm, shown as yellow dots, and compared with the control group, represented by black dots, in males (A) and females (B). Citrate synthase activity is depicted in green for males (C) and in orange for females (D). The comparison of relative citrate synthase activity in the control group between males and females is shown in (E). Data are presented as the mean ± S.D. (*n* = 2). Statistical analysis was performed using two-way analysis of variance (ANOVA) followed by multiple comparison tests for panels C and D, and an unpaired *t*-test for panel E. Significant differences from the respective control within each sex are indicated (*p < 0.05 vs. respective control). (For interpretation of the references to colour in this figure legend, the reader is referred to the web version of this article.)

Citrate synthase, an enzyme involved in the first step of the TCA cycle, was assessed to examine the effects of vinclozolin on mitochondrial metabolic function. Citrate synthase activity was assessed using the spectrophotometric method. Citrate synthase catalyzed the conversion of acetyl CoA and oxaloacetate to citrate and CoA-SH, after which CoA-SH reacted with DTNB to generate TNB, resulting in the change in absorbance at 412 nm. The change in absorbance of TNB mediated by citrate synthase was monitored and compared with the control in males (Fig. 1A) and females (Fig. 1B). Two-way ANOVA revealed a significant effect of vinclozolin concentration, with no significant effects of sex or interaction. The activity of citrate synthase remained unchanged up to 70 μM vinclozolin exposure but showed a significant decrease at 100 μM in both sexes. (Fig. 1C, D). Notably, no significant difference was confirmed in citrate synthase activity between male and female controls (Fig. 1E).

**Fig. 2:**
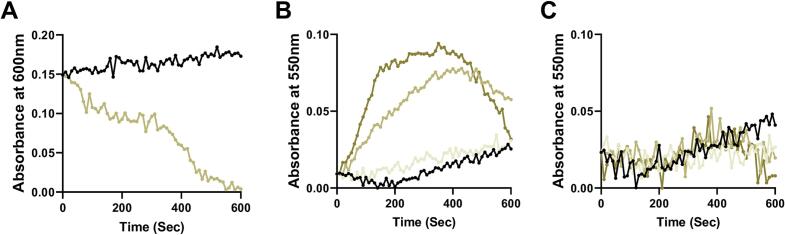
Absorbance interference of vinclozolin on substrates for mitochondrial complexes. Absorbance interference of vinclozolin on substrates for mitochondrial complexes I, II, III, and IV. The change in absorbance of DCPIP, as a substrate of complexes I and II, was monitored at 600 nm with or without 500 μM vinclozolin (A). The change in absorbance of oxidized cytochrome *c* (B) and reduced cytochrome *c* (C), as substrates of complexes III and IV, was monitored at 550 nm with or without 100, 200, and 300 μM vinclozolin. Vinclozolin-treated groups are represented by yellow dots, with deeper shades indicating higher concentrations, while the vinclozolin-untreated group is shown as black dots. (For interpretation of the references to colour in this figure legend, the reader is referred to the web version of this article.)

**Fig. 3:**
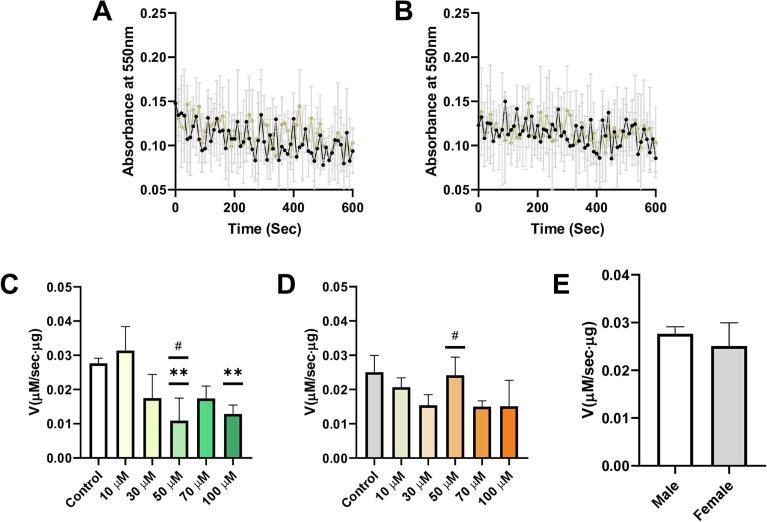
Mitochondrial complex IV activity after vinclozolin exposure on mouse liver. Mitochondrial complex IV activity of mouse liver after vinclozolin exposure. The change in absorbance of reduced cytochrome *c* was monitored at 550 nm following exposure to 100 μM vinclozolin, represented by yellow dots, and compared with the control group, shown as black dots, in males (A) and females (B). Mitochondrial complex IV activity is presented in green for males (C) and in orange for females (D). The comparison of relative mitochondrial complex IV activity in the control group between males and females is shown in (E). Data are presented as the mean ± S.D. (*n* = 3). Statistical analysis was performed using two-way analysis of variance (ANOVA) followed by multiple comparison tests for panels C and D, and an unpaired t-test for panel E. Significant differences from the respective control within each sex are indicated (**p < 0.01 vs. respective control), while sex differences at the same concentration are indicated (#p < 0.05 between males and females at the same concentration). (For interpretation of the references to colour in this figure legend, the reader is referred to the web version of this article.)

To assess the effect of vinclozolin on the electron transport chain, the activities of mitochondrial complexes I, II, III, and IV were measured. Prior to measuring mitochondrial complex activities, we confirmed whether vinclozolin itself caused any absorbance changes in the substrates of mitochondrial complexes. DCPIP used as a redox dye for complex I (CI) and complex II (CII) reactions, cytochrome *c* as the substrate for complex III (CIII), and reduced cytochrome *c* as the substrate for complex IV (CIV) were each exposed to vinclozolin, and the resulting changes in absorbance were monitored. While vinclozolin exposure altered the absorbance change of DCPIP and cytochrome *c*, the absorbance of reduced cytochrome *c* was not affected by vinclozolin (Fig. 2). Therefore, only the activity of complex IV was measured. The change in absorbance was monitored at 550 nm following exposure to 100 μM vinclozolin and compared to the control in males (Fig. 3A) and females (Fig. 3B). Two-way ANOVA revealed a significant effect of vinclozolin concentration and a significant interaction between sex and vinclozolin concentration, with no significant main effect of sex. In males, mitochondrial complex IV activity decreased significantly at 50 and 100 μM vinclozolin (Fig. 3C). However, although a slight reduction tendency was observed in females, the difference was not statistically significant compared with the control (Fig. 3D). Notably, a significant sex difference was observed at 50 μM vinclozolin, with males exhibiting lower CIV activity than females (Fig. 3C, D). Moreover, no significant difference was confirmed in mitochondrial complex IV activity between male and female controls (Fig. 3E).

**Fig. 4:**
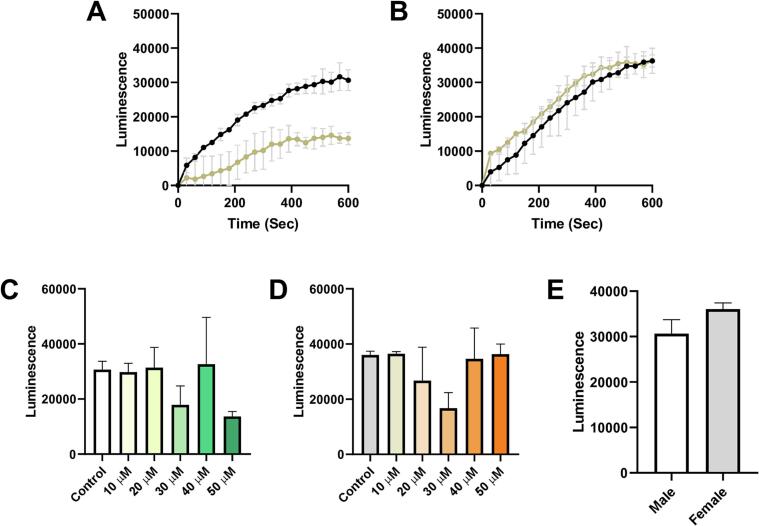
ATP production after vinclozolin exposure on mouse liver. ATP production after vinclozolin exposure. The luminescence change was monitored following exposure to 50 μM vinclozolin, represented by yellow dots, and compared with the control group, shown as black dots, in males (A) and females (B). Relative ATP production was represented in green for males (C) and in orange for females (D). The comparison of relative ATP production in the control group between males and females is shown in (E). Data are presented as the mean ± S.D. (n = 2). Statistical analysis was performed using two-way analysis of variance (ANOVA) followed by multiple comparison tests for panels C and D, and an unpaired t-test for panel E. No significant differences were observed. (For interpretation of the references to colour in this figure legend, the reader is referred to the web version of this article.)

ATP is produced through the proton gradient generated by the electron transport chain. ATP production was monitored by the luminescence of luciferin to measure the mitochondrial function in producing an energy source. In males, luminescence intensity decreased following exposure to 50 μM vinclozolin compared with the control (Fig. 4A), whereas no notable change was observed in females (Fig. 4B). Two-way ANOVA revealed no significant effects of vinclozolin concentration, sex, or their interaction. In terms of ATP production, vinclozolin exposure showed a slight decreasing tendency in males (Fig. 4C), but the change was not statistically significant, while no change was observed in females (Fig. 4D). Furthermore, ATP production did not differ significantly between male and female controls (Fig. 4E).

### 3.2 Vinclozolin increased antioxidant responses in females and promoted cytochrome *c* release

**Fig. 5:**
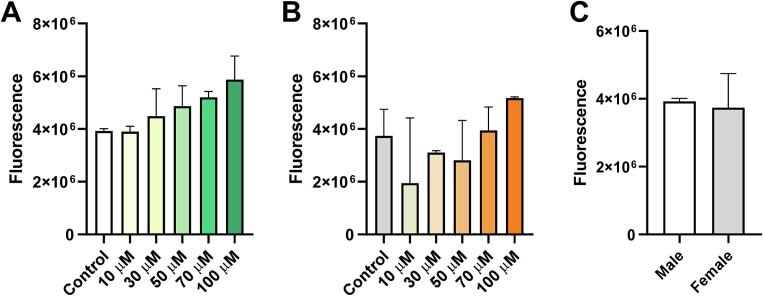
Relative ROS/RNS levels after vinclozolin exposure on mouse liver. Relative ROS/RNS levels after vinclozolin exposure. Fluorescence induced by ROS/RNS oxidation was measured in males (A) and females (B). The comparison of relative ROS/RNS levels in the control group between males and females is shown in (C). Data are presented as the mean ± S.D. (n = 3). Statistical analysis was performed using two-way analysis of variance (ANOVA) followed by multiple comparison tests for panels A and B, and an unpaired t-test for panel C. No significant differences were observed.

**Fig. 6:**
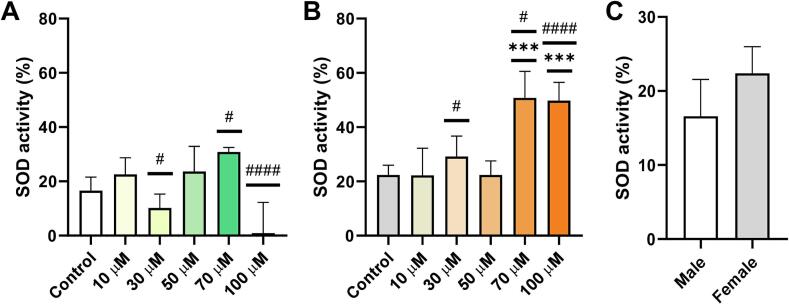
mSOD activity after vinclozolin exposure on mouse liver. mSOD activity after vinclozolin exposure. mSOD activity was determined in males (A) and females (B). The comparison of mSOD activity in the control group between males and females is shown in (C). Data are presented as the mean ± S.D. (n = 3). Statistical analysis was performed using two-way analysis of variance (ANOVA) followed by multiple comparison tests for panels A and B, and an unpaired t-test for panel C. Significant differences from the respective control within each sex are indicated (***p < 0.001 vs. respective control), while sex differences at the same concentration are indicated (#p < 0.05, ####*p* < 0.0001 between males and females at the same concentration).

**Fig. 7:**
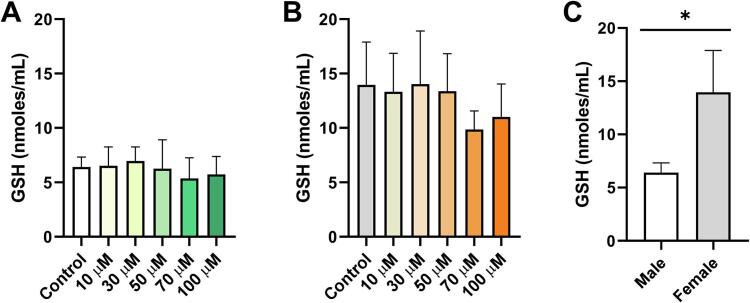
mGSH levels after vinclozolin exposure on mouse liver. mGSH levels after vinclozolin exposure. mGSH levels were determined in males (A) and females (B). The comparison of mGSH levels in the control group between males and females is shown in (C). Data are presented as the mean ± S.D. (n = 2). Statistical analysis was performed using two-way analysis of variance (ANOVA) followed by multiple comparison tests for panels A and B, and an unpaired t-test for panel C. Significant differences between male and female controls are indicated (*p < 0.05).

**Fig. 8:**
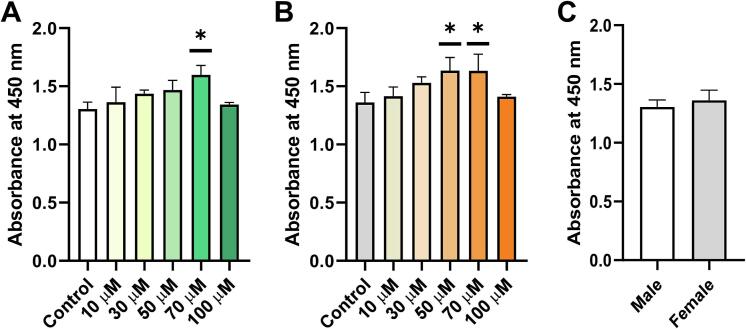
Cytochrome *c* release after vinclozolin exposure on mouse liver. Cytochrome *c* release after vinclozolin exposure. Cytochrome *c* release was determined in males (A) and females (B). The comparison of cytochrome *c* release in the control group between males and females is shown in (C). Data are presented as the mean ± S.D. (n = 2). Statistical analysis was performed using two-way analysis of variance (ANOVA) followed by multiple comparison tests for panels A and B, and an unpaired t-test for panel C. Significant differences from the respective control within each sex are indicated (*p < 0.05 vs. respective control).

To assess oxidative stress, ROS/RNS levels were measured following vinclozolin exposure. Two-way ANOVA revealed no significant effects of vinclozolin concentration, sex, or their interaction. In males, ROS/RNS levels showed an increasing tendency in a concentration-dependent manner, but the change was not statistically significant. (Fig. 5A). However, females exhibited slightly decreased levels of ROS/RNS at low vinclozolin concentrations, followed by an increase above 70 μM, but these changes were not statistically significant (Fig. 5B). Comparison of control values between males and females showed no significant difference (Fig. 5C). Antioxidants play a crucial role in regulating ROS/RNS generation. Oxidative stress can contribute to mitochondrial membrane permeabilization, leading to cytochrome *c* release and subsequent apoptosis. Therefore, to investigate the impact of vinclozolin on the antioxidant system, both enzymatic and non-enzymatic antioxidant activities were assessed. Mitochondrial SOD (mSOD) activity was conducted as an enzymatic system and mitochondrial GSH (mGSH) levels were measured as a non-enzymatic antioxidant system. In mSOD activity, two-way ANOVA revealed a significant effect of vinclozolin concentration and a significant interaction between sex and vinclozolin concentration, with no significant main effect of sex. In males, mSOD activity showed a slight increasing tendency up to 70 μM vinclozolin, followed by a decrease at 100 μM (Fig. 6A). In contrast, females exhibited a significant increase in mSOD activity at 70 and 100 μM concentrations (Fig. 6B). In addition, the results showed the significant differences between males and females were observed at 30, 70, and 100 μM concentrations (Fig. 6A, B). Furthermore, comparison of control values between males and females showed no significant difference (Fig. 6C). However, two-way ANOVA revealed no significant effects of vinclozolin concentration, sex, or their interaction on mGSH levels. Consistently, mGSH levels were not altered following vinclozolin exposure in both sexes (Fig. 7A, B). Notably, the control values of mGSH levels were significantly higher in females than in males (Fig. 7C). To examine whether vinclozolin affect mitochondrial membrane permeabilization, cytochrome *c* release assay was conducted. Two-way ANOVA revealed a significant effect of vinclozolin concentration on mitochondrial cytochrome *c* release, with no significant effects of sex or interaction. Consistently, mitochondrial cytochrome *c* release increased significantly at 70 μM in males, and at 50 and 70 μM in females following vinclozolin exposure (Fig. 8A, B). However, cytochrome *c* release did not show any significant difference between male and female controls (Fig. 8C).

## 4 Discussion

Endocrine disruptors (EDs) affect multiple organs—including the liver, pancreas, and adipose tissue—by disturbing cellular metabolism through mitochondrial dysfunction (Nadal et al., 2017). Among these, the liver plays a central role in regulating energy metabolism, signaling pathways, and insulin sensitivity, all of which are closely associated with the development of metabolic diseases. Hepatic mitochondria are essential for maintaining hepatocyte homeostasis by providing energy and regulating key metabolic processes (Morio et al., 2021). Given these roles, we hypothesized that vinclozolin, an endocrine disruptor, targets hepatic mitochondria, resulting in mitochondrial impairment and subsequent hepatic dysfunction. Since exposure of the liver to EDs has been linked to glucose intolerance and insulin resistance, both major features of metabolic diseases such as type 2 diabetes (Marroqui et al., 2018), elucidating the effects of vinclozolin on hepatic mitochondria may provide insights into its potential contribution to the pathogenesis of metabolic disorders.

In this study, we examined the effects of direct exposure to vinclozolin on mitochondria isolated from mouse liver. Vinclozolin exposure impaired mitochondrial function and was associated with the increasing tendency of oxidative stress, indicating mitochondrial dysfunction. Specifically, vinclozolin reduced citrate synthase activity in both males and females, while complex IV activity was decreased only in males, suggesting difference of mitochondrial function between sexes. Reduced activity of citrate synthase may suggest early alterations in mitochondrial metabolic capacity. Previous studies have shown that vinclozolin exposure decreased key TCA metabolites—including citrate, α-ketoglutarate, and fumarate—in the striped marsh frog (*Limnodynastes peronei*), which are associated with oxidative stress-related metabolic alterations (Borrás et al., 2003). While we did not directly measure TCA intermediates, the reduction in citrate synthase activity observed in the present study may be consistent with mitochondrial alterations induced by vinclozolin exposure. However, these findings do not directly demonstrate impaired TCA cycle flux or broad disruption of energy metabolism in hepatic mitochondria. Citrate synthase activity was reduced in both sexes, but the decline in complex IV activity occurred only in males, indicating a sex-dependent difference in the mitochondrial response to vinclozolin.

Despite the decrease in citrate synthase and complex IV activities, ATP production was not significantly changed by vinclozolin exposure. Notably, bioluminescence generated by the luciferin–luciferase reaction used for ATP measurement could not be detected at vinclozolin concentrations above 70 μM. However, a slight decreasing tendency in ATP levels was observed in males, which is consistent with the reduction in complex IV activity observed only in males. Similarly, ROS/RNS levels also showed an increasing tendency primarily in males. This result may be associated with impaired electron transport, as reduced complex IV activity can promote electron leakage and partial oxygen reduction during oxidative phosphorylation (Devaux et al., 2020). These sex-dependent results may be explained by differences in mitochondrial antioxidant capacity. In the present study, mitochondrial antioxidant responses differed between sexes. Specifically, SOD activity was significantly increased in females following vinclozolin exposure, whereas mGSH levels did not show significant changes in either sex, although basal levels were significantly higher in females. These findings suggest that females may have a greater capacity to counteract oxidative stress due to both enhanced antioxidant responses and higher baseline antioxidant levels. Previous studies have also reported that females generally exhibit stronger antioxidant defense systems and reduced susceptibility to mitochondrial oxidative damage compared to males (Ventura-Clapier et al., 2017; Torrens-Mas et al., 2020). These findings suggest that female mitochondria may be more resistant to vinclozolin-induced mitochondrial stress, whereas males may be more vulnerable to disruptions in mitochondrial function.

Increased ROS can induce cytochrome *c* release through mitochondrial membrane permeability transition pores or via activation of BAX proteins, ultimately leading to apoptosis (Garrido et al., 2006). Although ROS/RNS levels did not show statistically significant increases, cytochrome *c* release was increased in both males and females following vinclozolin exposure, indicating mitochondrial membrane disruption and the initiation of apoptotic signaling. Cytochrome *c* release may be triggered not only by oxidative stress but also by structural and functional impairment of the mitochondrial electron transport chain. Previous studies have suggested that mitochondrial dysfunction, including impaired electron transport and loss of membrane integrity, can induce cytochrome *c* release independently of increases in ROS levels (Kroemer et al., 2007; Wang and Youle, 2009). Therefore, the observed increase in cytochrome *c* release in this study may reflect direct mitochondrial damage caused by vinclozolin.

This study was conducted using isolated mitochondria, which allows the direct assessment of mitochondrial toxicity but does not fully reflect physiological exposure conditions in vivo. Therefore, the findings of this study may not explain the full toxicological mechanisms that occur at the cellular or organism level. Nevertheless, this study provides important insight into the intrinsic hepatic mitochondrial toxicity of vinclozolin and highlights sex-dependent differences by directly assessing its effects on isolated mitochondria, independent of systemic and cellular regulation. Furthermore, these results suggest that vinclozolin-induced mitochondrial dysfunction could contribute to metabolic disorders such as insulin resistance and type 2 diabetes.

## 5 Conclusion

Vinclozolin is a widely used fungicide known to act as an ED. Mitochondrial dysfunction has been associated with metabolic diseases such as insulin resistance and type 2 diabetes, and ED exposure in the liver has also been linked to these metabolic disorders. In addition, EDs are known to impair mitochondrial activity. Thus, elucidating the effects of vinclozolin on hepatic mitochondria is essential for understanding its potential contribution to metabolic disorders. In this study, vinclozolin was directly treated to isolated mitochondria from mouse liver to evaluate its acute effects on mitochondrial function, independent of systemic endocrine signaling. The results showed that vinclozolin induced mitochondrial dysfunction by impairing citrate synthase and complex IV activities and disrupting mitochondrial membrane integrity. Furthermore, significant sex-dependent differences were observed, with males exhibiting more prominent mitochondrial impairment. These sex-dependent differences are likely attributed to intrinsic differences in antioxidant capacity between males and females. Therefore, this study highlights the importance of considering sex differences in research on EDs, even when evaluating their direct mitochondrial effects. Overall, our findings demonstrate that vinclozolin can directly induce mitochondrial dysfunction, suggesting its potential to cause metabolic disorders through impaired mitochondrial energy metabolism and membrane integrity. Moreover, by revealing sex-dependent mitochondrial responses to vinclozolin, this study advances the understanding of mechanisms underlying the sex-dependent impact of environmental toxicants.

## Author contributions

Hwayeon Lim: Investigation, Analyses, Visualization, Validation, Writing-Original draft. Jisun Choi: Writing-Reviewing and Editing, Analyses, Visualization, Validation. Joo Young Huh, Hoonsung Cho, and Sooim Shin: Conceptualization, Supervision, Writing-Reviewing and Editing. All the authors contributed to the study and the manuscript. If the manuscript is accepted for publication, I agree to transfer all copyright ownership of the manuscript to the Current Research in Toxicology, which covers the rights to use, reproduce, or distribute the article.

## Funding

This research was supported by Basic Science Research Program through the National Research Foundation of Korea (NRF) funded by the 10.13039/501100002701Ministry of Education (RS-2025-25411631), and by the “Regional Innovation System & Education (RISE)” through the Gwangju RISE Center, funded by the Ministry of Education (MOE) and the Gwangju Metropolitan Government, Republic of Korea (2025-RISE-05-011).

## Declaration of competing interest

The authors declare the following financial interests/personal relationships which may be considered as potential competing interests: Sooim Shin reports financial support was provided by National Research Foundation of Korea. Sooim Shin reports financial support was provided by Regional Innovation System & Education (RISE) - Gwangju RISE Center. Reports a relationship with that includes:. Has patent pending to. If there are other authors, they declare that they have no known competing financial interests or personal relationships that could have appeared to influence the work reported in this paper.
